# Synthesis and Improved Photoresponse of Silicon Nanoparticle Heterostructures

**DOI:** 10.3390/nano16070411

**Published:** 2026-03-29

**Authors:** Jun Wang, Hrilina Ghosh, Siva Sivoththaman

**Affiliations:** Department of Electrical and Computer Engineering, University of Waterloo, Waterloo, ON N2L 3G1, Canada; j872wang@uwaterloo.ca (J.W.); sivoththaman@uwaterloo.ca (S.S.)

**Keywords:** silicon nanoparticles, heterostructures, surface modification, photoresponse

## Abstract

In this paper, we have synthesized silicon nanoparticles (SiNPs) via a simple, scalable hydrothermal method using [3-(2-aminoethylamino)propyl] trimethoxysilane (AEAPTMS) as the Si precursor and L-ascorbic acid (L-AA) as the reductant. In order to improve carrier transport in the synthesized NPs to enhance their applicability in optoelectronic devices, a surface modification process had been carried out to replace the original long-chain dehydroascorbic acid (DHA) ligand with a shorter-chain 3-mercaptopropionic acid (MPA) ligand. A hybrid test structure was then fabricated composed of the surface-modified SiNP layer with a conductive polymer, PEDOT:PSS, which served as the hole transport layer. This SiNP-PEDOT:PSS planar heterostructure served as a platform to probe the photoresponse and carrier dynamics of the modified nanoparticles. Compared to the as-synthesized SiNPs, the surface-modified SiNPs achieved a 20% increase in carrier lifetime and an on/off ratio of 7.28 at ±1 V applied bias under UV illumination. These findings highlight the potential of SiNPs for integration into solution-processed optoelectronic devices.

## 1. Introduction

Semiconductor nanoparticles (NPs) have fundamentally advanced the field of solution-processed optoelectronics, offering size-tunable bandgaps, high surface-to-volume ratio, controllable light emission and facile thin-film fabrication [1,2,3]. These unique properties have led to the rapid development of next-generation light-emitting diodes, solar cells and photodetectors [4]. Historically, the highest-performing nanomaterial-based devices have relied on heavy-metal chalcogenides, such as cadmium selenide and lead sulfide. Despite their mature development, the inherent toxicity of lead and cadmium restricts their use in consumer-facing and eco-friendly technologies [5]. This demands a strong need for exploring non-toxic solutions.

In this context, silicon nanoparticles (SiNPs) have attracted significant research interest as a ‘green’, non-toxic alternative with the advantages of elemental abundance, biocompatibility and a tunable bandgap suitable for broad spectral absorption [6]. Additionally, the possibility of simple, scalable synthesis routes increases the commercial viability of SiNPs. In contrast to traditional methods such as electrochemical etching, template synthesis, and plasma synthesis, which typically require high energy inputs or hazardous corrosive reagents like hydrofluoric acid, hydrothermal synthesis utilizes relatively low temperatures and mild precursors. This makes the process significantly more cost-effective and sustainable [7,8]. In addition to ensuring good optoelectronic properties in the synthesized SiNPs, ensuring their long-term stability is also of great importance. SiNPs have the inherent problem of getting oxidized when exposed to moisture or air [9,10].

‘Green’ hydrothermal synthesis routes are highly effective at producing water-soluble, stable nanoparticles. However, they typically yield particles capped with bulky, insulating ligands. These native ligands, while necessary for colloidal stability, create significant interparticle tunneling barriers that impede charge carrier transport [11,12]. Consequently, as-synthesized SiNPs often exhibit poor conductivity in solid-state films, limiting their utility in optoelectronic devices where efficient charge extraction is required [13].

To address this limitation, surface engineering is essential. The substitution of long-chain ligands with short-chain ones can effectively reduce the interparticle distance and passivate surface defects, thereby enhancing charge transport [14]. To further improve carrier transport and overall device performance, hybrid heterostructures have also garnered attention. By coupling SiNPs with conductive polymers such as poly(3,4-ethylenedioxythiophene):poly(styrenesulfonate) (PEDOT:PSS), we can exploit the advantages of both materials: the strong optical absorption of the SiNPs and the excellent hole-transporting capability of the polymer [15,16,17]. The selective carrier extraction mechanism minimizes interfacial recombination and allows for the sensitive detection of photogenerated carriers, making the planar heterostructure an ideal testbed for evaluating SiNP quality.

In this work, we present a facile and environment-friendly hydrothermal method to synthesize SiNPs using [3-(2-aminoethylamino)propyl] trimethoxysilane (AEAPTMS) as the Si precursor and L-ascorbic acid (L-AA) as the reductant [18]. The synthesis is then followed by a solution-based surface modification process using 3-mercaptopropionic acid (MPA) as the ligand, which substitutes the initial long carbon chain dehydroascorbic acid (DHA) ligand and passivates the SiNP surface, thereby facilitating improved device performance. A hybrid test structure was then fabricated composed of the surface-modified SiNP layer with a conductive polymer, PEDOT:PSS. This SiNP-PEDOT:PSS planar heterostructure served as a platform to probe the photoresponse and carrier dynamics of the modified nanoparticles. Our results indicate that the surface-modified SiNPs exhibit superior optoelectronic performance compared to the as-synthesized SiNPs, achieving a 20% increase in carrier lifetime and a distinct on/off response under UV illumination. These findings highlight the potential of SiNPs for integration into solution-processed optoelectronic devices.

## 2. Materials and Methods

### 2.1. Synthesis of Silicon Nanoparticles

Synthesis of SiNPs was carried out inside a three-neck round-bottom glass flask under an inert atmosphere maintained using nitrogen (N_2_) gas throughout the reaction. The reaction flask was mounted onto a stirring heating mantle. An RF coil stirring controller with a magnetic stirrer and a proportional–integral–derivative controller were used to regulate stirring and heating, respectively. The heating mantle was filled with silica beads to ensure even heat distribution around the base of the flask, with the top of the flask bundled in synthetic insulation. The three necks of the flask were used for precursor injection and extraction, thermocouple insertion for temperature monitoring, and N_2_ flow. The reaction setup was designed to contain the reaction within the flask, thus minimizing contamination and exposure.

For the synthesis, 2 mL [3-(2-aminoethylamino) propyl] trimethoxysilane (AEAPTMS, 98%) was injected into the flask as the Si precursor. It was mixed with 8 mL ethanol and deionized (DI) water as solvent to start the hydrolysis reaction. The mixture was stirred until a homogeneous solution was obtained. Then, 0.450 g L-ascorbic acid (L-AA, 99%) was injected as the reducing agent and the temperature was gradually increased. Six different temperatures (25 °C, 35 °C, 45 °C, 55 °C, 65 °C, 75 °C) were investigated in order to optimize the process conditions for synthesis. At each temperature, the reaction proceeded for 25 min, after which the reaction was stopped and the product extracted. The resulting solution was purified by dialysis for 24 h, followed by filtration using a 20 nm pore-size filter. The SiNPs solution thus obtained was stored in a refrigerator for further use and testing.

Photoluminescence (PL) spectroscopy (Edinburg Instruments, Livingston, UK) with a 450 W xenon lamp as the excitation light source was used to measure the PL emission of the synthesized SiNPs. All PL measurements were done at an excitation wavelength of 395 nm. The chemical composition of the SiNPs was studied by X-ray photoelectron spectroscopy (XPS) (VG ESCALab 250, Thermo Fisher, Waltham, MA, USA).

### 2.2. Formation of Silicon Nanoparticle Heterostructures

In order to test the photoresponse of the synthesized SiNPs, a surface modification step was performed prior to film formation. The as-synthesized SiNPs were functionalized by a solution-based ligand exchange process. The solvent during SiNP synthesis was changed to ethanol to better dissolve MPA in the SiNP solution and aid in film formation. Then, 0.5 mL 3-mercaptopropionic acid (MPA, 99%) ligand solution was added to 2 mL SiNP solution. The mixture was sealed and sonicated for 60 min for complete ligand substitution and to prevent aggregation. As the MPA ligands attached to available sites on the surface of the SiNPs and replaced the original DHA ligands, the color of the solution gradually changed from pale yellow to orange. The SiNPs before and after surface modification are denoted as DHA-SiNPs and MPA-SiNPs, respectively.

After the surface modification, the SiNP-PEDOT:PSS heterostructure films were formed on indium tin oxide (ITO)-coated glass substrates of 22 mm × 22 mm in size. The substrates were sequentially cleaned using acetone, isopropyl alcohol and DI water. The ITO probe contact area was defined using Kapton masking tape. Next, poly (3,4-ethylenedioxythiophene)—poly (styrenesulfonate) (PEDOT:PSS, 1.3 wt% dispersion in water) was spin-cast as the hole transport layer. This was followed by spin-casting the SiNPs as the photoactive layer. In order to demonstrate the need for surface modification, both MPA-SiNPs and DHA-SiNPs were used in two different sets of devices. All other fabrication steps were identical for both sets of test structures. After spin-coating the SiNPs, the samples were annealed at 180 °C for 5 min to remove residual solvent and improve interfacial adhesion. Finally, the top electrode was formed by electron beam evaporation (IntlVac Nanochrome I, Georgetown, ON, Canada) of a 250 nm thick silver (Ag) film using an aligned shadow mask.

To characterize successful SiNP surface modification, Fourier transform infrared (FTIR) spectroscopy (Vertex 70 V, Bruker, Ettlingen, Germany) was performed. PL spectroscopy was performed and the fluorescence lifetime of the DHA-capped and MPA-capped SiNPs was measured using time-resolved spectroscopy with a pulsed LED (EPLED-380, Livingston, UK 375 nm, 5 MHz, pulsewidth: 943.3 ps), and the fluorescence decay curve was analyzed by the time-correlated single-photon counting (TCSPC) method. The absorption of the SiNPs was measured using UV–visible spectroscopy (Shimadzu, Kyoto, Japan). Cyclic voltammetry (CV) was performed using a CHI600E electrochemical analyzer (CH Instruments, Austin, TX, USA) to determine the highest occupied molecular orbital (HOMO) and lowest unoccupied molecular orbital (LUMO) values. The measurement employed a three-electrode system with platinum working and counter electrodes and an Ag/AgCl reference electrode, using 0.1 M tetrabutylammonium hexafluorophosphate as the electrolyte. Ferrocene with known E_HOMO_ = −4.8 eV was used as the reference material. The current–voltage (I-V) characteristics of the test structures were measured with an Agilent 4155C semiconductor parameter analyzer (Axiomtest, Vista, CA, USA) connected to a 2-point-probe station in a sealed black chamber to prevent unwanted ambient light. For UV-illuminated I-V measurements, a Cole Parmer 4-Watt UV lamp (Vernon Hills, IL, USA) with dual 365 nm wavelength tubes was mounted directly over the device under test and illuminated at full intensity.

## 3. Results and Discussion

### 3.1. Characterization of As-Synthesized SiNPs

The optical properties and chemical composition of the as-synthesized SiNPs were characterized using PL spectroscopy and XPS, respectively.

#### 3.1.1. Photoluminescence Properties of Synthesized SiNPs

**Figure 1:**
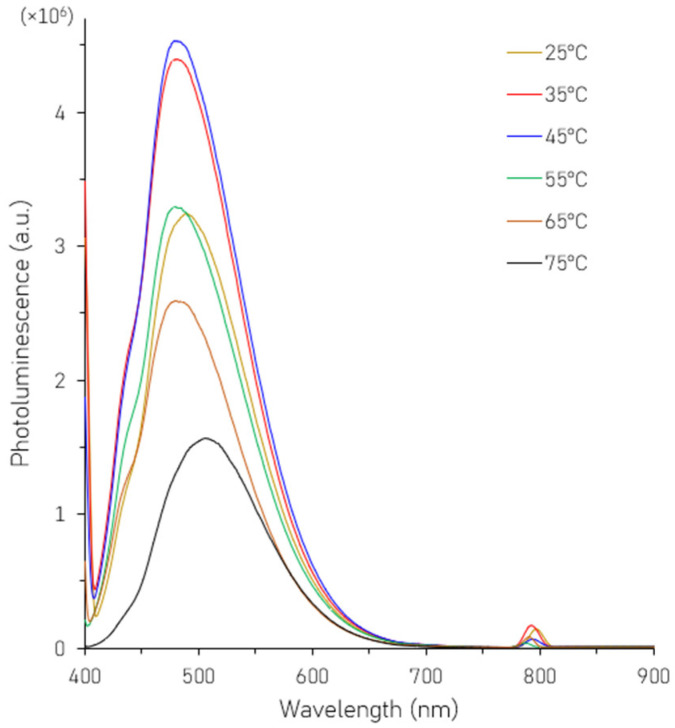
Photoluminescence (PL) spectra of SiNPs synthesized under different temperature conditions.

The PL spectra of SiNPs synthesized at different reaction temperatures are shown in Figure 1. Temperatures higher than 75 °C are not investigated as L-AA, which is a mild reducing agent, starts to degrade from 85 °C. From the figure, it is observed that the highest PL intensity with the peak center at around 484 nm occurs for SiNPs grown at 45 °C. For higher temperatures, the growth rate of the nanoparticles accelerates, leading to a larger average particle size, causing a redshift in the PL peak position, due to an increase in the nanoparticle size. Additionally, at higher temperatures, the PL intensity also decreases, possibly because of rapid nanoparticle growth and more surface defects. On the other hand, at lower temperatures, the thermal energy required to overcome the activation barrier for the hydrolysis of AEAPTMS and subsequent reduction by LAA is insufficient. Consequently, the formation of the silicon crystalline core and the Si-O-Si network is incomplete, leading to a low density of high-emission NPs. Thus, 45 °C is selected as the optimum temperature and all subsequent characterization and testing is performed on SiNPs synthesized at 45 °C.

#### 3.1.2. Chemical Composition of Synthesized SiNPs

**Figure 2:**
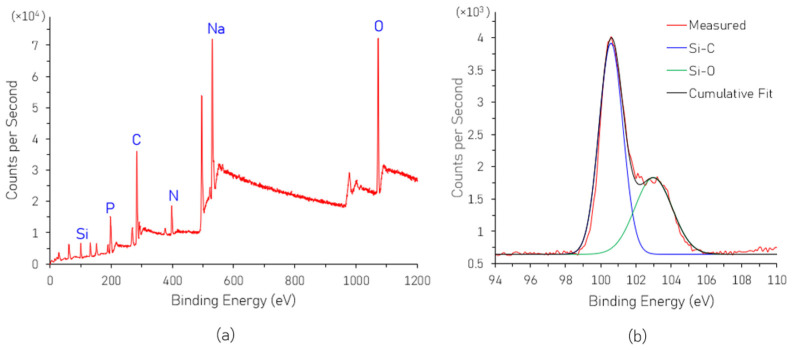
(**a**) XPS spectra of the as-synthesized SiNPs; (**b**) deconvoluted XPS spectrum of Si 2p orbital.

The XPS survey spectra of the as-synthesized SiNPs are shown in Figure 2a. The characteristic peaks at binding energies of 101 eV, 198 eV, 284 eV, 398 eV, 530.5 eV, and 1072 eV in Figure 2a correspond to elements Si, P, C, N, Na, O, respectively. Na and O originate from the dialysis buffer solution. The composition of the SiNPs can be analyzed from the deconvoluted XPS spectrum of the Si 2p orbital shown in Figure 2b. From the figure, the binding energies at 100.2 eV and 102.6 eV are attributed to Si-C and Si-O, respectively [19]. In the AEAPTMS Si precursor used for SiNP synthesis, the ratio of Si-O:Si-C is 3:1. However, that ratio in the synthesized SiNPs is 0.7, which is a significant decrease. The possible mechanism for this is that the Si precursor hydrolyzes during which -OCH3 groups are substituted by -OH groups. This is followed by condensation, which forms the Si-O-Si network. After adding the reductant L-AA, the silicon oxide network is restored and forms the Si core, and a DHA ligand attaches on the SiNP surface [20]. Thus, the decreasing amount of Si-O compared to that in the original Si precursor confirms the reduction process.

### 3.2. Characterization of Surface-Modified SiNPs

The effect of surface modification on the SiNPs was characterized using FTIR, PL spectroscopy, UV-Vis spectroscopy and fluorescence lifetime analysis.

#### 3.2.1. FTIR Analysis

**Figure 3:**
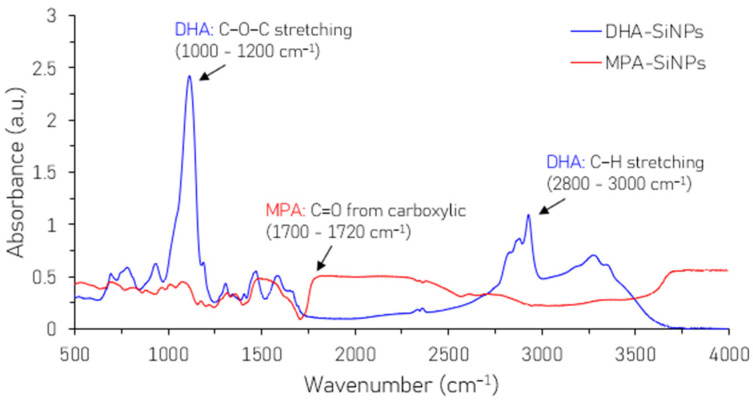
FTIR spectra of SiNPs before and after surface modification.

FTIR was used to analyze the chemical changes due to surface modification. The FTIR spectra of the SiNPs before and after ligand exchange are shown in Figure 3. In Figure 3, the vibration in 2800–3000 cm^−1^ corresponds to the C-H stretching in DHA-SiNPs, which originates from long hydrocarbon chains. A clear decrease in C-H stretching is evident in MPA-SiNPs because of the shorter ligand length. In the DHA-SiNP spectrum, we also note a sharp peak at ~1000–1200 cm^−1^ which is characteristic of the C-O stretching vibrations of DHA. In the MPA-SiNP spectrum, this peak is almost suppressed. Moreover, a new absorption feature appears in the MPA-SiNP spectrum at ~1700–1720 cm^−1^, which can be attributed to the C=O stretching vibration of the carboxylic acid group in the MPA ligand. Therefore, in the MPA-SiNP spectrum, the attenuation of the C-H and C-O vibrations and the emergence of the C=O vibration confirm the successful attachment of the MPA ligands to the SiNPs.

#### 3.2.2. Emission/Absorbance Analysis

**Figure 4:**
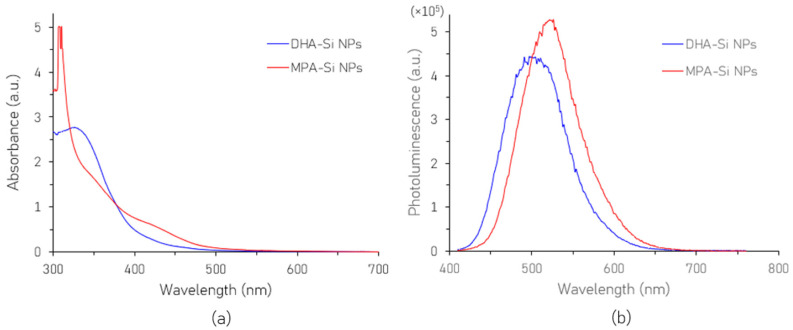
UV–visible absorbance spectra (**a**) and PL emission spectra (**b**) of SiNPs before and after surface modification.

Figure 4a represents the UV–visible absorption spectra of the SiNPs before and after surface modification. Both samples exhibit strong absorption in the UV wavelengths, characteristic of silicon nanoparticles. The as-synthesized DHA-SiNPs exhibit a broad absorption peak at around 330 nm. In contrast, the absorption peak of the MPA-SiNPs is blueshifted to around 310 nm and is noticeably sharper, indicating a relatively narrow size distribution.

The PL emission spectra of the as-synthesized DHA-SiNPs and the surface-modified MPA-SiNPs are presented in Figure 4b. From the figure, it is observed that the MPA-SiNPs show a higher intensity, indicating superior surface passivation which is also evidenced by the enhanced lifetime of the MPA-SiNPs. In addition to the intensity enhancement, the PL peak of the MPA-SiNPs is redshifted. The covalent bond between the thiol (-SH) group of the MPA ligand and the SiNP surface is stronger than the bond between the oxygen-based groups of the original DHA ligand and the SiNP surface. This stronger covalent bond may lead to electron delocalization which may mimic a slightly larger particle size, resulting in a redshift of the PL peak intensity [21].

#### 3.2.3. Lifetime Analysis

**Figure 5:**
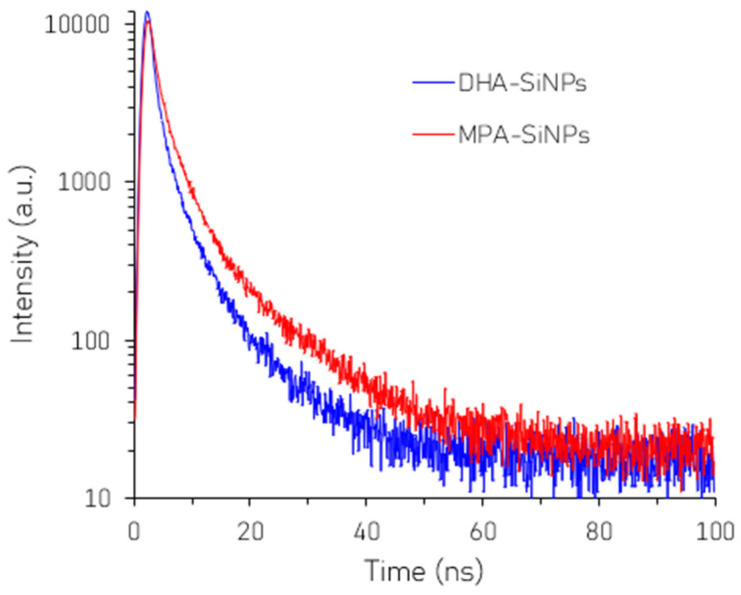
Time-resolved fluorescence decay curves of SiNPs before and after surface modification.

Figure 5 presents the PL decay curves of the SiNPs before and after surface modification. The corresponding fluorescence lifetimes, extracted by analyzing the decay curves by the time-correlated single-photon counting (TCSPC) method, were 1.04 ns and 1.26 ns for DHA-SiNPs and MPA-SiNPs respectively, indicating that the lifetime increased by around 20% after surface modification. The 20% increase in fluorescence lifetime of the surface-modified MPA-SiNPs indicates that MPA passivates surface defects more effectively than the control DHA ligands. The thiol (-SH) group in MPA effectively passivates surface states and dangling bonds. This results in reduced non-radiative recombination and a longer lifetime compared to the control DHA.

### 3.3. Photoresponse of SiNP-PEDOT:PSS Heterostructure Films

**Figure 6:**
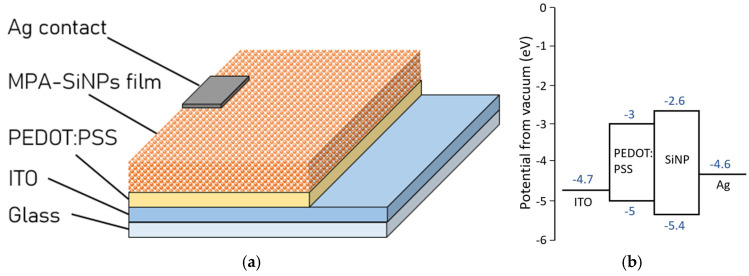
(**a**) Schematic of the fabricated test structures along with the (**b**) energy band diagram.

Figure 6a shows the schematic of the test structures fabricated with SiNP-PEDOT:PSS heterostructure films. The structure consists of ITO-coated glass as the substrate followed by a layer of PEDOT:PSS, MPA-SiNPs and finally Ag as the top electrode. We employed PEDOT:PSS as the hole transport layer (HTL) because of its high conductivity and suitable work function (about 5.0 eV) [22,23]. The shallow band of PEDOT:PSS can effectively transfer holes from MPA-SiNPs as well as block electron injection and recombination. The corresponding band structure of the device is shown in Figure 6b. The energy levels used for ITO, Ag and PEDOT:PSS are from the literature [24] and that of the MPA-SiNP film was calculated from cyclic voltammetry and a UV-Vis absorbance spectrum. The bandgap (E_g_) of ~2.8 eV for MPA-SiNPs was determined from the UV-Vis absorption spectrum (Figure 4a) by the Tauc plot method, based on the relation ${\left(\alpha h\nu \right)}^{1/n}=A(h\nu -E_{g})$. By applying an exponent of n = ½, plotting ${\left(\alpha h\nu \right)}^{2}$ against photon energy $\left(h\nu \right)$ and extrapolating the linear portion of the absorption edge to the *x*-axis where ${\left(\alpha h\nu \right)}^{2}=0$, the bandgap was determined to be approximately 2.8 eV. The HOMO level was determined via CV using ferrocene with E_HOMO_(ferrocene) = −4.8 eV as the reference. From CV measurements, the onset potential (E_OX_) was 0.6 eV. Hence, the HOMO level for the SiNPs was calculated as E_HOMO_ = −[−E_HOMO_(ferrocene) + E_OX_] = −[4.8 + 0.6] eV = −5.4 eV. From the HOMO level of −5.4 eV and the bandgap of ~2.8 eV, the LUMO level was calculated as E_LUMO_ = E_HOMO_ + E_g_ = (−5.4 + 2.8) eV = −2.6 eV, as we can see in Figure 6b. In order to illustrate the need for surface modification, test structures with DHA-SiNPs were also fabricated using an identical structure and process.

**Figure 7:**
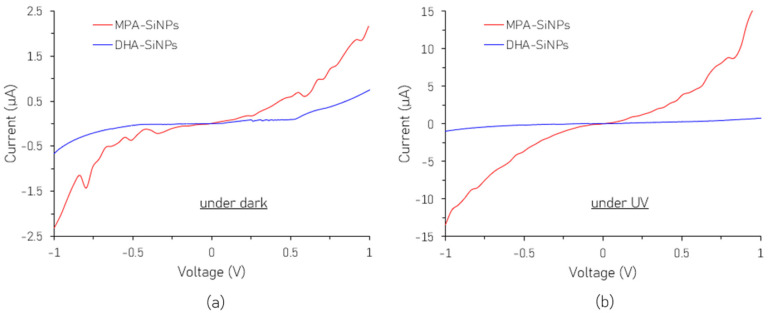
I-V characteristics of DHA-SiNPs and MPA-SiNPs (**a**) in the dark and (**b**) under UV illumination.

The current–voltage curves of both sets of devices in the dark and under UV illumination are shown in Figure 7. Ten test devices on a single substrate were measured over several days with negligible variation in the I-V characteristics, thus ensuring reproducibility. The devices were also subjected to repeated dark and UV illumination cycles and remained stable without any observable degradation. From Figure 7a, we can see that MPA-SiNPs have a sharp enhancement in dark current response compared to DHA-SiNPs. The reduction in ligand length from a six-carbon chain for DHA to a three-carbon chain for MPA reduces the interparticle spacing. As the charge hopping rate between nanoparticles increases exponentially with reducing interparticle distance, charge transport and conductivity improve for MPA which is evidenced by the increased dark current. From Figure 7b, it can be noted that, while both devices responded to light, the photocurrent was significantly higher for the MPA-SiNP device than that for the DHA-SiNP device. Further, at an applied bias of ±1 V, the on–off (photocurrent to dark current) ratio for the DHA-SiNP and MPA-SiNP devices is about 1.04 and 7.28 respectively. This clearly demonstrates the superior photoresponse of the MPA-SiNP device.

The poor light response of the DHA-SiNPs can be attributed to the adverse carrier transportation caused by the long side chain which causes most of the photogenerated carriers to recombine before being collected by the electrode. In contrast, while functionalizing the SiNPs’ surface with the MPA ligands, there was better surface passivation and reduced ligand length, which contributed to improved carrier transport and collection.

**Figure 8:**
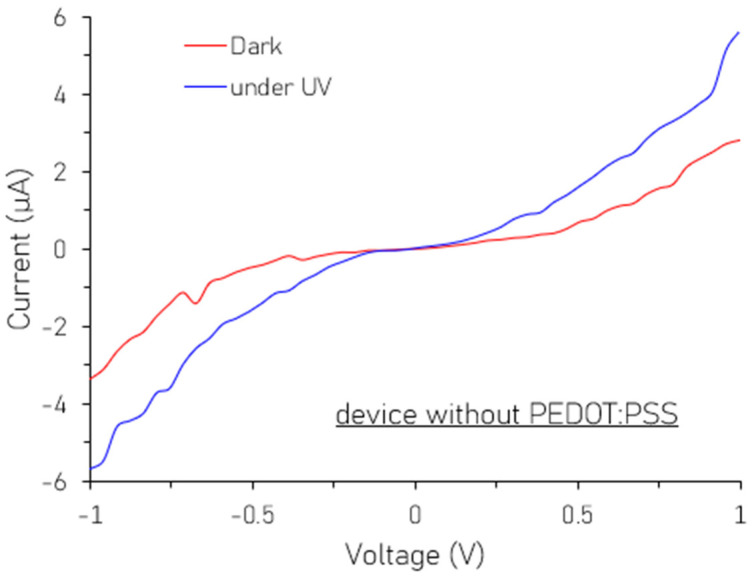
I-V characteristics of SiNPs without PEDOT:PSS.

To further understand the role of PEDOT:PSS in our test structure, we present the I-V characteristics of a control device consisting of a simple ITO/MPA-SiNP/Ag structure, without the PEDOT:PSS layer, in Figure 8. A direct comparison between the I-V characteristics of our standard device (ITO/PEDOT:PSS/MPA-SiNP/Ag) in Figure 7 and our control device in Figure 8 reveals that the inclusion of PEDOT:PSS leads to a significant increase in the photocurrent by about an order of magnitude. Moreover, the on/off ratio for the control device is significantly lower at 2 compared to the 7.28 achieved with a PEDOT:PSS layer. This enhancement suggests that the PEDOT:PSS layer provides a selective pathway for photogenerated holes while effectively reducing recombination.

## 4. Conclusions

In conclusion, SiNPs have been produced from AEAPTMS and L-AA using a facile, inexpensive hydrothermal method. The synthesized SiNPs exhibited strong PL emission. For optoelectronic applications, along with the quality of the NPs, the ligand attached on the NP surface plays a key role in device performance. Long-chain ligands hinder carrier transport. Hence, we have deployed a solution-based surface modification process to replace the original 6C DHA ligand with a shorter 3C MPA ligand. Successful ligand exchange has been confirmed by FTIR absorbance, lifetime analysis and UV–visible absorbance analysis. The fluorescence lifetime of the surface-modified MPA-SiNPs was improved by 20% compared to DHA-SiNPs. Following the surface modification process, test devices were fabricated with SiNP-PEDOT:PSS heterostructures where SiNP was the active light-harvesting layer and the PEDOT:PSS aided in hole transport. The device with MPA-SiNPs showed strong response to UV illumination with an on/off ratio of 7.28 at an applied voltage of ±1 V. This demonstrates the high potential of the non-toxic MPA-SiNP–PEDOT:PSS heterostructures for application in optoelectronic devices.

## Data Availability

The original contributions presented in this study are included in the article. Further inquiries can be directed to the corresponding author.
